# The effects of alpha‐pinene against paracetamol‐induced liver damage in male rats

**DOI:** 10.14814/phy2.70227

**Published:** 2025-02-04

**Authors:** Kaveh Rahimi, Anahita Rezaie, Younes Allahverdi, Parham Shahriari, Mahtab Taheri Mirghaed

**Affiliations:** ^1^ Department of Basic Sciences, Faculty of Veterinary Medicine Shahid Chamran University of Ahvaz Ahvaz Iran; ^2^ Department of Pathobiology, Faculty of Veterinary Medicine Shahid Chamran University of Ahvaz Ahvaz Iran

**Keywords:** alpha pinene, liver, paracetamol, rat

## Abstract

This study aims to evaluate the hepatoprotective effect of alpha‐pinene against N‐acetyl‐p‐aminophenol, paracetamol, (APA)‐induced liver damage in rats. Thirty Wistar rats were divided into five groups (*n* = 6): Group 1: Normal (control). Group 2: APA 640 mg/kg. Group 3: alpha‐pinene 50 mg/kg (APA+ αPi 50 mg/kg). Group 4: alpha‐pinene 100 mg/kg (APA+ αPi 100 mg/kg). Group 5: silymarin 50 mg/kg (APA+ SIL). Alpha‐pinene or silymarin was orally administered after APA administration for 14 consecutive days. This study investigated liver damage by preparing pathology slides from liver tissue. Levels of AST, ALT, ALP, total bilirubin, total antioxidant capacity (TAC), and total oxidant status (TOS) were measured. Inflammatory factors, including NF‐kB gene expression and levels of IL‐6 and TNF‐a, were also measured. Administering alpha‐pinene with APA can prevent liver damage induced by APA. Alpha‐pinene can enhance TAC while reducing TOS, ALT, AST, ALP, and total bilirubin. Moreover, the results have also revealed that alpha‐pinene decreases NF‐kB expression, which leads to a reduction in IL‐6 and TNF‐a levels. It appears that alpha‐pinene induces liver protective effects against APA damage by reducing the activity of liver enzymes, improving antioxidant/oxidative status, and reducing inflammation through the regulation of NF‐kB and pro‐inflammatory cytokines.

## 1 INTRODUCTION

Liver disease is a global concern with potential serious impacts on public health and drug efficacy. Complementary and alternative medicines are increasingly considered for treating acute and chronic liver diseases (Ahsan et al., 2009). Nonsteroidal anti‐inflammatory drugs (NSAIDs) are FDA‐approved drugs used to treat muscle pain, dysmenorrhea, arthritic conditions, fever, gout, and migraines. They are also used as opioid‐sparing agents in certain acute trauma cases (Dawood, 2006; Oyler et al., 2015; Phillips & Currier, 2004; Shekelle et al., 2017). N‐acetyl‐p‐amino‐phenol (paracetamol, acetaminophen) (APA) is widely used to treat pain and fever globally. However, it can cause dose‐related liver damage, leading to nearly 500 deaths annually in the United States. Additionally, it results in about 100,000 calls to US Poison Control Centers, 50,000 emergency room visits, and 10,000 hospitalizations per year (Lee, 2017).

The exact molecular mechanism of APA‐induced liver injury has not been fully explained yet. At therapeutic concentrations, around 60%–90% of APA is metabolized in the liver through glucuronidation and sulfation, with a small portion (approximately 5%–15%) being metabolized by the cytochrome P450 pathway (CYP450) (Kalsi et al., 2011; Marto et al., 2021). Under overdose conditions, more APA is converted to N‐acetyl‐p‐benzoquinone imine (NAPQI) by cytochrome P450 enzymes (Weiqiao Chen et al., 1998). NAPQI oxidizes the thiol groups of proteins and generates reactive oxygen species (ROS) (Iorga et al., 2017). APA increases total antioxidant status (TAC) and total oxidant status (TOS) in the liver. ROS directly harms the lipids, proteins, enzymes, and DNA of liver cells and can also trigger immune‐mediated oxidative damage (Villanueva‐Paz et al., 2021). It is crucial to evaluate the overall level of oxidative stress in the body, for instance, by assessing TAC and TOS. Interestingly, APA‐induced cell necrosis results in increased production of proinflammatory cytokines, which worsen liver inflammation (McGill & Jaeschke, 2014; Saeedi Saravi et al., 2016).

Pinene (C10H16) is a terpenoid hydrocarbon found in nature, particularly in pine essential oils. It has two isomers, alpha and beta, each with two enantiomers, yielding four active isomers. Alpha‐pinene is oil‐ and ethanol‐soluble, while beta‐pinene is oil‐soluble. They are used in various applications including bakery products and candy production. Pinenes can be produced through biotransformation and undergo rearrangement and ring‐opening reactions (Berger, 2007; Erman & Kane, 2008; Vespermann et al., 2017; Winnacker, 2018). Pinenes are natural compounds with diverse biological activities, such as flavoring agents, fungicides, fragrances, and antiviral and antimicrobial properties. They are also used in the synthesis of high‐quality polymers. Pinenes are generally recognized as safe and have a good safety profile, allowing their use in various chemicals (Almirall et al., 1996; Bakhtazad et al., 2024; da Silva Rivas et al., 2012; Satoh et al., 2014). Recently, various studies have been conducted regarding the therapeutic effects of alpha‐pinene. Alpha‐pinene effectively reduces pain response in the formalin test (Rahimi, Shirvani, et al., 2023; Rahimi, Zalaghi, et al., 2023). It modulates tumor necrosis factor‐alpha (TNF‐a) and interleukin‐1β (IL‐1β) and down‐regulates cyclooxygenase 1 (COX‐1) protein expression in the spinal cord. Alpha‐pinene also decreases TNF‐α, IL‐1β, and malondialdehyde (MDA) while increasing superoxide dismutase (SOD), glutathione (GSH), and catalase (CAT) levels at the site of formalin injection in rats (Rahimi, Zalaghi, et al., 2023). Pretreatment with alpha‐pinene has been found to be effective in reducing ethanol‐induced gastric damage by regulating Nrf2/HO‐1. When alpha‐pinene was administered before ethanol, gastric mucosa damage was reduced, and ulcer inhibition percentage increased (Rahimi, Shirvani, et al., 2023). In a study conducted, the inhibitory effects of alpha‐pinene on liver cancer cells have been investigated both in vitro and in vivo (W. Chen et al., 2015). Pine needle oil has shown a significant reduction in the growth of hepatocellular carcinoma cells (Chen et al., 2015).

Numerous studies have demonstrated the role of antioxidant compounds in liver damage (Li et al., 2015). However, there is a lack of information about the impact of alpha‐pinene on liver damage. Therefore, this study aims to investigate the effect of alpha‐pinene against liver damage caused by APA and the possible mechanisms involved in this process.

## 2 MATERIALS AND METHODS

### 2.1 Animals

Male Wistar rats with an average weight of 200 ± 20 g were kept in a standard research setup. They had access to pellet food (KTPO Company, Iran), water, and natural daylight hours (cycle: dark and light). Before conducting research, the rats were accustomed to the laboratory environment for a week. The study was conducted in accordance with ARRIVE guidelines (Percie du Sert et al., 2020). The study protocol was approved by the Ethics Committee of the Faculty of Veterinary Medicine, Shahid Chamran University of Ahvaz, Ahvaz, Iran (IR.SCU.REC.1402.054).

### 2.2 Experimental design

Rats were obtained from the Laboratory Animal Care Center, Faculty of Veterinary Medicine, Shahid Chamran University, Ahvaz. A total of 30 Wistar rats (10 weeks old) were divided into five groups with six rats each: Group 1: Normal (control). Group 2: APA 640 mg/kg (Jalinous Pharmaceutical Co., Iran) (Islam et al., 2021) (dissolved in 0.9% NaCl). Group 3: alpha‐pinene 50 mg/kg (APA+ αPi 50 mg/kg). Group 4: alpha‐pinene 100 mg/kg (APA+ αPi 100 mg/kg). Group 5: silymarin (Zardband Pharmaceutical Co., Iran) 50 mg/kg (APA+ SIL). Samples were collected from the animals 24 h after the final administration of APA.

Except for the control group, which was given 0.9% NaCl, all groups received APA orally for 14 days. All administrations were given between 09:30 a.m. and 10:30 a.m. Alpha‐pinene (% minimum by GLC 97.18, Refractive index in 20 C 1.464–1.468) was provided by Saghez Sazi Kurdistan Manufacturing Co. (Van), Iran (CAS No. 7785‐26‐4, FEMA No. 2902). In this study, the silymarin used was derived from milk thistle extract, according to the manufacturer's description.

### 2.3 Evaluation AST, ALT, ALP, and total bilirubin

A biochemical analyzer (Hitachi, Tokyo, Japan) was used to measure alanine aminotransferase (ALT) (ZistChem, Iran), aspartate aminotransferase (AST) (ZistChem, Iran), alkaline phosphatase (ALP) (Delta‐dp, Iran), and total bilirubin (Man Company, Iran) levels in the serum.

### 2.4 Evaluation body and liver weights

The body weight of the animals was measured before and after the experiment, as well as the weight of the livers.

### 2.5 Evaluation of total antioxidant capacity (TAC) levels

The Ferric Reducing Ability of Plasma (FRAP) assay involves reducing the ferric‐tripyridyltriazine (Fe3 + ‐TPTZ) complex to ferrous tripyridyltriazine (Fe2 + ‐TPTZ) using antioxidants in a sample at low pH. The end product (Fe2 + ‐TPTZ) exhibits a blue color with a maximum absorption at 593 nm. The increase in absorbance is directly linked to the antioxidant capacity of the plasma. FRAP reagent, which includes 300 mM acetate buffer, pH = 3.6, 10 mM TPTZ solution in hydrochloric acid 40 mM, 20 mM solution of iron chloride hexahydrate (FeCl3.6H2O), and 100–1000 molar ascorbic acid standard solution, was added to 10 μL of the sample (homogenized liver tissue). The initial absorbance was measured at a wavelength of 593 nm. The samples were then incubated at a temperature of 37 degrees Celsius for 5 min, and the optical absorption of the solution at a wavelength of 593 nm was measured again using a microplate reader. In order to determine the protein concentration in the samples, we utilized the Bradford method. To determine the TAC, we used the following formula:

FRAP value of Sample (μM) = (Change in absorbance of sample from 0 to 5 min/Change in absorbance of standard from 0 to 5 min) × FRAP value of standard (1000 μM) (Benzie & Strain, 1999).

### 2.6 Evaluation of total oxidant status (TOS) levels

To assess the TOS, we utilized a method developed by Erel (2005). In this approach, the oxidant present in the sample is responsible for oxidizing the ferrous‐o‐ranitidine complex to ferric. The ferric ion produced in the sample then interacts with xylenol orange in an acidic environment, forming a colored complex that can be measured at a wavelength of 560 nm. The standard used in this method is H_2_O_2_, and the results are reported in umol H_2_O_2_ equiv/L. In order to normalize the data, we took into account the amount of protein present in the sample (Erel, 2005).

### 2.7 Real‐time PCR

RNA was extracted using the RNA Extraction Kit (Parstous, Iran) to analyze the expression of nuclear factor kappa B (NF‐κB). A commercial kit was used for cDNA synthesis, which was performed using random hexamer primers (Parstous, Iran). The cDNA samples were then evaluated through real‐time PCR reactions using the real‐time kit. Real‐time PCR was performed in 20 L of total reaction mixture containing SyberGreen (SYBR® Green‐ Parstous, Iran), and each primers (forward and reverse) (Table 1). All samples were run twice; each reaction included negative control samples without cDNA and RNA control samples.

**TABLE 1:** Primers sequences.

Gene name	Sequence	Lenght bp	GeneBank ACC
GAPDH‐rat‐F	AGTTCAACGGCACAGTCAAG	119	NM_017008.4
GAPDH‐rat‐R	TACTCAGCACCAGCATCACC		
NFKB‐rat‐F	TCAACATGGCAGACGACGAT	134	NM_001276711.1
NFKB‐rat‐R	TTGAAGGTATGGGCCATCTGT		

### 2.8 Histopathological evaluation

The liver tissues were fixed using 10% buffered formalin for histopathological studies. The tissues were then trimmed (to 5 μm thickness) using a microtome and embedded in paraffin wax. The tissue sections were stained with hematoxylin and eosin, then photographed using an Olympus DP 72 microscope in Tokyo, Japan.

The liver damage score was classified according to injury severity, using a numerical scale from 0 to 3, with 0 indicating no damage and 3 representing the most severe injury.

### 2.9 Evaluation of IL‐6 and TNF‐a levels

Biochemical parameters such as IL‐6 (Karmaniapasrgene, Iran) and TNF‐α (Kiazist, Hamedan, Iran) were measured in the liver. The samples were homogenized in 500 μL of lysis buffer and then centrifuged at 11,000 ***g*** for 15 min at 4°C. The supernatants were diluted with a diluent buffer and added to each well of the ELISA kits. The total protein content was measured by the Bradford method.

### 2.10 Statistical analysis

The data presented in this study are expressed as mean ± standard deviation (SD). The normality of the data was assessed using the Kolmogorov–Smirnov test. Statistical analysis was conducted using T test–paired and one‐way analysis of variance (ANOVA) with Tukey's multiple comparisons in GraphPad Prism software (version 8). A significance level of *p* < 0.05, *p* < 0.01, and *p* < 0.001 was considered for the analysis.

## 3 RESULTS

### 3.1 Pathological findings

**FIGURE 1:**
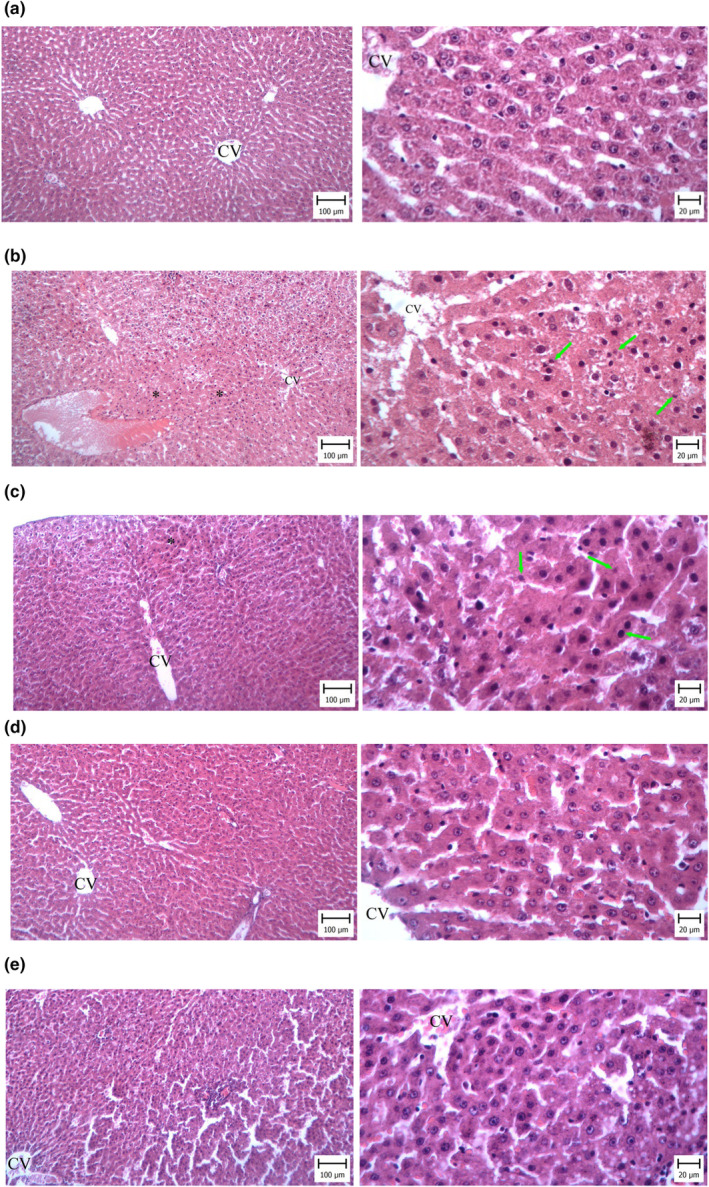
Pathological findings. (a) Control. Normal liver structure is observed (hematoxylin and eosin staining (X100 and X400)). (b) APA. Pay attention to the necrosis of liver hepatocytes (star). These cells can be recognized with a more colorful cytoplasm, and the nuclei of these cells show various changes such as pyknosis and karyolysis (green arrow). (c) APA + aPi 50. Necrosis (star sign) is seen with less severity than in the APA group. Cells also show nuclear changes such as pyknosis and karyochexia. (d) APA + aPi 100. Necrosis and infiltration of inflammatory cells are not seen. (e) APA + SIL. The liver structure appears normal.

In the microscopic examination of liver sections, we observed that the group receiving APA showed focal necrosis of hepatocytes. This type of necrosis was coagulation‐type, characterized by darkening of the liver cell cytoplasm and changes in the nucleus such as pyknosis, karyorrhexis, and karyolysis. Additionally, we noticed several areas where inflammatory cells had infiltrated the liver. Similarly, but to a lesser extent, the group receiving APA + aPi 50 exhibited liver damage similar to the APA group. However, in the APA + aPi 100 group, we found less severe liver damage with fewer instances of hepatocyte necrosis and no inflammatory cell presence. Notably, we did not observe any pathological damage in the control group or the group receiving APA + SIL (Figure 1).

**FIGURE 2:**
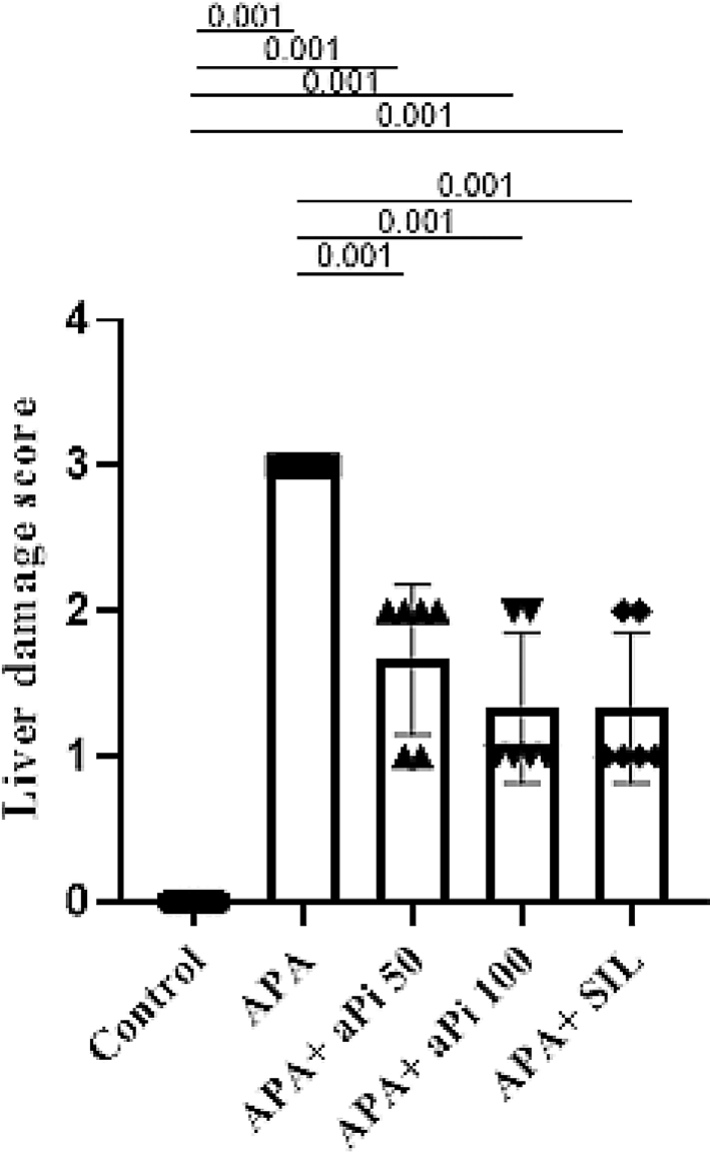
Effects of aPi on the liver damage score in APA‐treated rat liver. All values are expressed as mean ± SD (*n* = 6).

The groups treated with APA, APA + aPi 50, APA + aPi 100, and APA + SIL had a significantly higher tissue damage score compared to the control group (*p* < 0.001). Conversely, the groups treated with APA + aPi 50 mg/kg, APA + aPi 100 mg/kg, and APA + Sil 50 mg/kg had a significantly lower tissue damage score compared to the PAR group (*p* < 0.001) (Figure 2).

### 3.2 AST, ALT, ALP, and Total bilirubin

The groups treated with APA, APA + aPi 50, APA + aPi 100, and APA + SIL had a significantly higher average AST, ALT, ALP, and total bilirubin compared to the control group. Conversely, the groups treated with APA + aPi 50, APA + aPi 100, and APA + SIL had a significantly lower average AST, ALT, ALP, and total bilirubin compared to the APA group (Table 2 shows the averages and level of significance).

**TABLE 2:** Effect of api treatment on blood serum levels of AST, ALT, ALP, and total bilirubin.

	Control	APA	APA+ αPi 50	APA+ αPi 100	APA+ SIL
ALT u/l	51.57 ± 0.45	145.9 ± 7.21^###^	124.8 ± 2.45^###,*^	126.2 ± 0.91^###,*^	127.7 ± 1.60^###,*^
AST u/l	183.3 ± 0. 51	354.8 ± 36.93^###^	250.9 ± 1.68**	245.2 ± 5.58**	262.2 ± 1.76^#,*^
ALP u/l	190.5 ± 1.50	556.0 ± 33.16^##^	339.2 ± 31.32*	372.2 ± 60.12^#,*^	319.7 ± 32.35**
Total bilirubin mg/dL	0.44 ± 0.06	10.54 ± 0.50^###^	8.16 ± 0.45^###,*^	7.31 ± 0.35^###,**^	8.44 ± 0.60^###,*^

*Note*: All values are expressed as mean ± SD (*n* = 6). Different letters indicate the level of significance between distinct groups. ^#^ *p* < 0.05, ^##^ *p* < 0.01, ^###^ *p* < 0.001 compared to control group; *p < 0.05, **p < 0.01 compared to APA group.

### 3.3 Body weight and liver weight

The results showed that there was no significant difference between the study groups in body weight before and after the study. Also, there was no significant difference in liver weight between the different groups (Table 3).

**TABLE 3:** Effect of api treatment on body and liver weight.

	Control	APA	APA+ αPi 50	APA+ αPi 100	APA+ SIL
Body weight (day 0)	221.7 ± 6.80	218.00 ± 15.72	225.00 ± 5.00	215.00 ± 8.54	127.7 ± 1.60
Body weight (day 14)	241.00 ± 8.71**	225.7 ± 18.01*	245.3 ± 3.78**	235.3 ± 10.07**	234.00 ± 9.16***
Liver weight	10.13 ± 0.20	9.90 ± 0.51	10.16 ± 0.99	9.98 ± 0.05	10.11 ± 0.60

*Note*: All values are expressed as mean ± SD (*n* = 6). Different letters indicate the level of significance between distinct groups. *p < 0.05, **p < 0.01, ***p < 0.001 significant differences in weight changes in different groups at the end of the study compared to the beginning of the study.

In the control group, the animals' body weight at the end of the study was significantly higher than it was at the beginning (*p* < 0.01). Additionally, the APA group also showed a significant increase in body weight from the start to the end of the study (*p* < 0.05). Similarly, in groups APA + aPi 50, APA + aPi 100, and APA + SIL, the body weight recorded at the conclusion of the study was significantly greater than at the beginning. (*p* < 0.01, *p* < 0.01, and *p* < 0.001, respectively).

### 3.4 TAC and TOS levels in the liver

**FIGURE 3:**
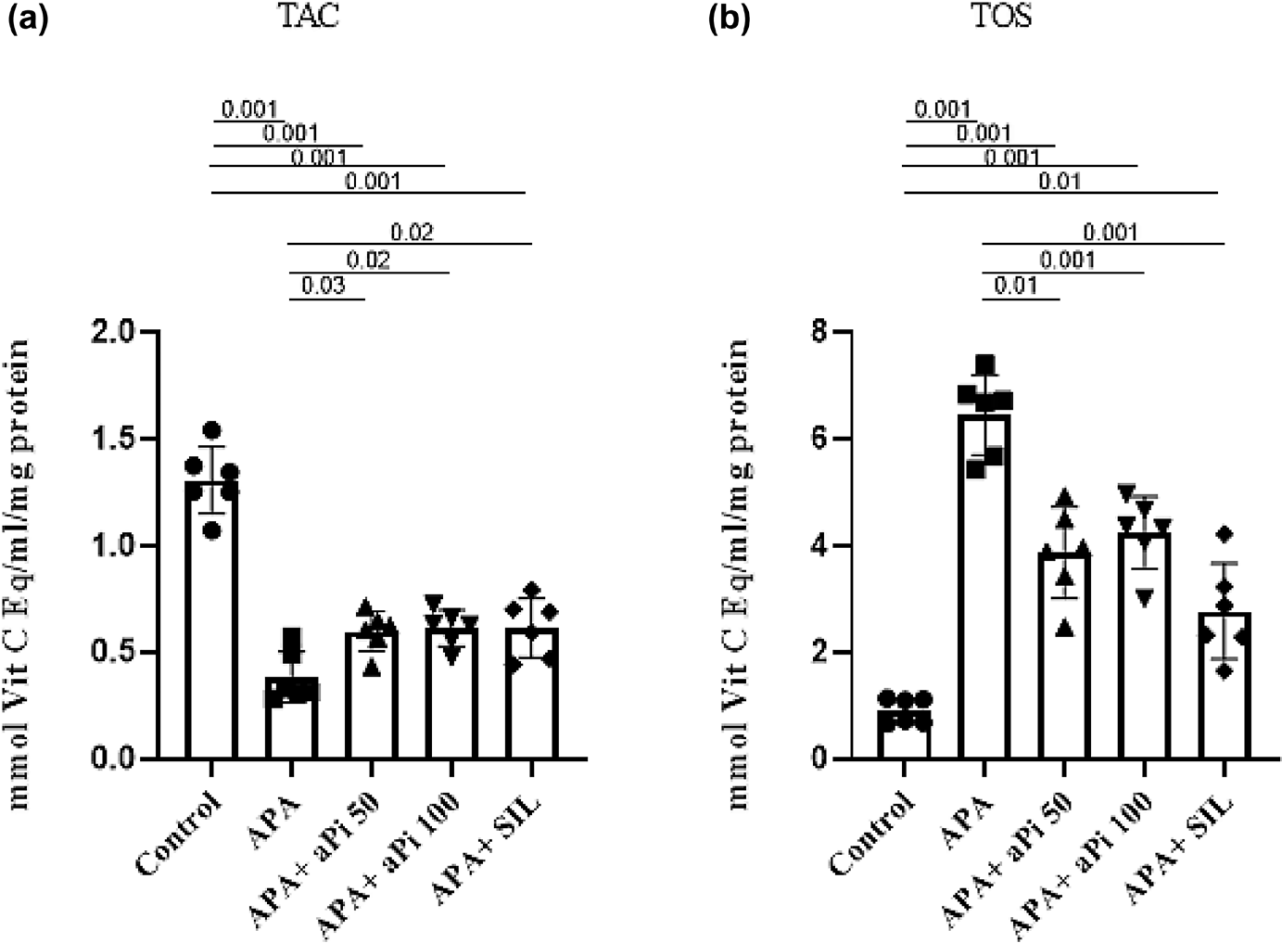
Effects of aPi on the TAC (a) and TOS (b) in APA‐treated rat liver. All values are expressed as mean ± SD (*n* = 6).

According to the results of this study, it was found that the groups treated with APA (0.38 ± 0.11), APA + aPi 50 (0.59 ± 0.09), APA + aPi 100 (0.61 ± 0.08), and APA + SIL (0.61 ± 0.13) had a significantly lower average TAC compared to the control group (1.30 ± 0.15) (*p* < 0.001). Conversely, the groups treated with APA + aPi 50, APA + aPi 100, and APA + SIL had a significantly higher average TAC compared to the APA group (*p* < 0.05). This can be seen in Figure 3a.

According to the results of this study, it was found that the groups treated with APA (6.45 ± 0.75), APA + aPi 50 (3.85 ± 0.85), APA + aPi 100 (4.23 ± 0.67), and APA + SIL (2.76 ± 0.89) had a significantly higher average TOS compared to the control group (0.90 ± 0.23) (*p* < 0.001, *p* < 0.001, *p* < 0.001, and *p* < 0.01, respectively). Conversely, the groups treated with APA + aPi 50, APA + aPi 100, and APA + SIL had a significantly lower average TOS compared to the APA group (*p* < 0.01, *p* < 0.001, and *p* < 0.001, respectively). This can be seen in Figure 3b.

### 3.5 NF‐kB expressions in the liver

**FIGURE 4:**
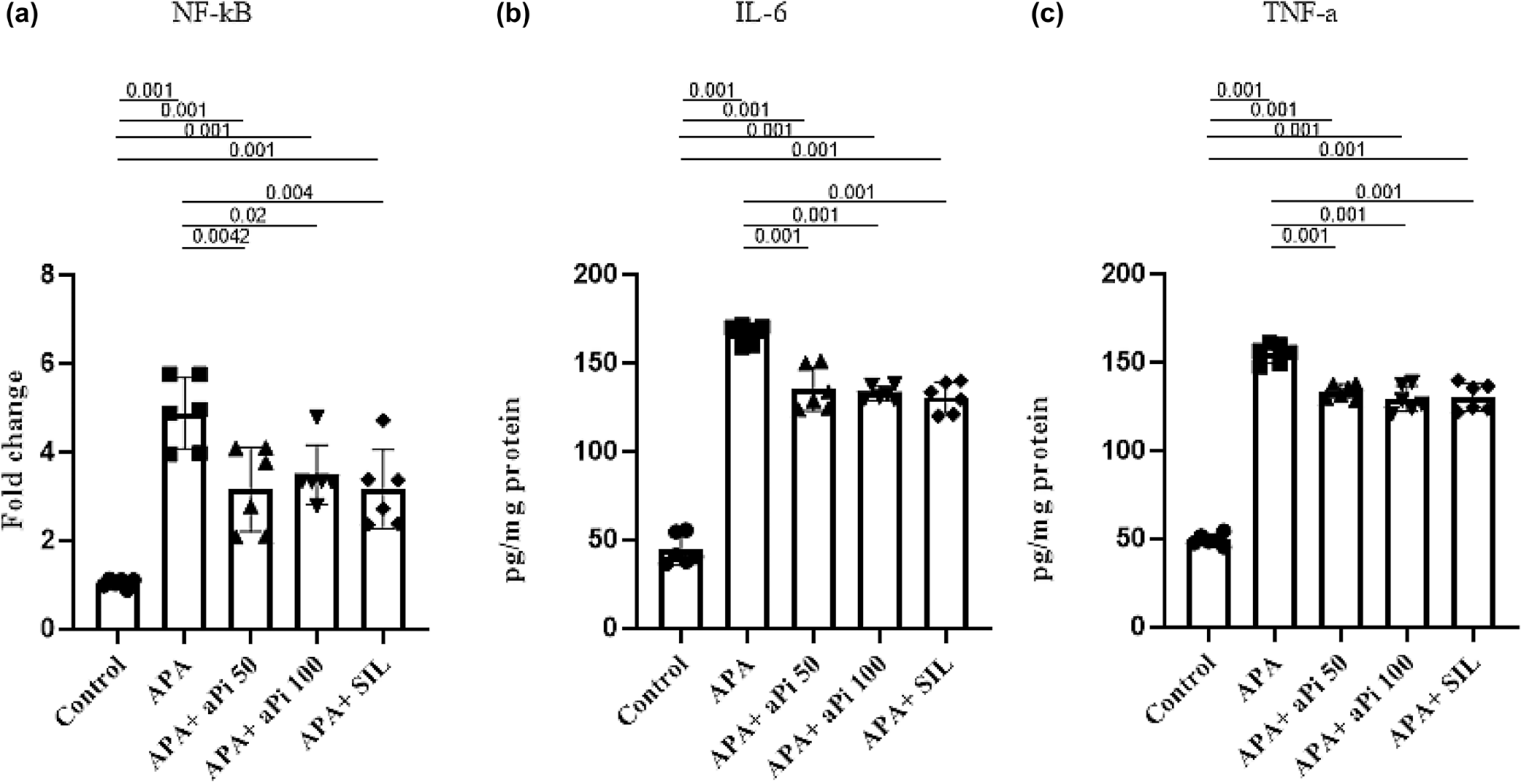
Effects of aPi on the mRNA expression of NF‐kB (a) and IL‐6 (b) and TNF‐a (c) levels in APA‐treated rat liver. All values are expressed as mean ± SD (*n* = 6).

The groups that received APA (4.88 ± 0.80), APA + aPi 50 (3.15 ± 0.95), APA + aPi 100 (3.48 ± 0.67), and APA + SIL (3.15 ± 0.89) had significantly higher NF‐kB levels than the control group (1.04 ± 0.09) (*p* < 0.001). However, the groups that received APA + aPi 50, APA + aPi 100, and APA + SIL treatments had significantly lower NF‐kB levels than the APA group (*p* < 0.01, *p* < 0.05, and *p* < 0.01, respectively). (Figure 4a).

### 3.6 IL‐6 and TNF‐a levels in the liver

The groups that received APA (166.7 ± 5.55), APA + aPi 50 (135.4 ± 12.27), APA + aPi 100 (133.00 ± 3.89), and APA + SIL (130.6 ± 8.66) had significantly higher IL‐6 levels than the control group (44.39 ± 8.45) (*p* < 0.001). However, the groups that received APA + aPi 50, APA + aPi 100, and APA + SIL treatments had significantly lower IL‐6 levels than the APA group (*p* < 0.001) (Figure 4b).

The groups that received APA (155.00 ± 5.47), APA + aPi 50 (133.5 ± 4.05), APA + aPi 100 (129.40 ± 7.26), and APA + SIL (130.3 ± 7.86) had significantly higher TNF‐a levels than the control group (49.45 ± 3.16) (*p* < 0.001). However, the groups that received APA + aPi 50, APA + aPi 100, and APA + SIL treatments had significantly lower TNF‐a levels than the APA group (*p* < 0.001) (Figure 4c).

## 4 DISCUSSION

In mice, acetaminophen (APAP) hepatotoxicity can be induced rapidly with a single dose. Due to its clinical relevance and experimental convenience, APAP intoxication has become a widely used model for studying liver injury. Early studies showed that rats are resistant to APAP toxicity, leading researchers to prefer mice for mechanistic studies. However, new findings from the last years encourage a reevaluation of the differences between these species. This comparison could provide valuable insights and determine whether rats could be a viable option for APAP studies. To investigate this further, a survey was conducted in which both rats and mice were treated with APAP, and several parameters were measured, including liver injury, APAP metabolism, oxidative stress, and the activation of c‐Jun N‐terminal kinase (JNK). Consistent with previous data, the study found that rats resisted APAP toxicity. Nevertheless, APAP metabolism was similar in both species (McGill et al., 2012). In our research, we used an extended period of 14 days to induce liver injury in rats with APAP. Our findings confirm the establishment of a liver injury model using APAP in rats.

Our research suggests that alpha‐pinene, given together with APA, can prevent liver damage caused by APA. Alpha‐pinene raises TAC levels while lowering TOS, AST, ALT, ALP, and total bilirubin. Moreover, our findings indicate that alpha‐pinene reduces NF‐kB expression, which leads to a decrease in IL‐6 and TNF‐a levels.

Drug‐induced liver injury (DILI) refers to unintended liver damage caused by commonly used drugs, often under‐recognized or under‐diagnosed (Devarbhavi et al., 2021). There are two types of DILI: intrinsic and idiosyncratic. Intrinsic DILI is dose‐dependent and predictable, while idiosyncratic DILI (IDILI) is not dose‐dependent and is associated with specific medications. APA is the most typical drug causing intrinsic DILI (Andrade et al., 2019; Russo et al., 2004; Tujios & Fontana, 2011). DILI can be categorized into hepatocellular, cholestatic, and mixed liver injury based on its characteristics. Hepatocellular injury is the most common type and is characterized by hepatocyte necrosis, mild cholestasis, and elevated enzyme levels (AST, ALT, gamma‐glutamyl transpeptidase (GGT), and ALP). The cholestatic injury involves cholestasis in the bile ducts and elevated GGT and ALP levels. Mixed liver injury presents features of both hepatocellular and cholestatic injury (Larrey, 2000; Zimmerman, 2000). Our results indicated that APA leads to increased levels of AST, ALT, and ALP. Pathological findings also revealed the presence of liver tissue necrosis.

DILI can be acute or chronic. Chronic DILI is defined as the failure to return to previous of bilirubin level, liver enzymes, and other indications of progressive liver disease (such as portal hypertension, hepatic ascites, coagulation abnormalities, and hepatic encephalopathy) within 6–9 months after the onset. Around 13.6% of all DILI cases are chronic, and 15%–20% of acute cases can become chronic. Cholestatic DILI is more likely to become chronic than hepatocellular DILI (Chalasani et al., 2015; Chalasani et al., 2021).

5%–9% of APA is metabolized by cytochrome enzymes, mainly by CYP2E1, converted to NAPQI (Lancaster et al., 2015). NAPQI is an active metabolite detoxified by rapid binding to GSH, which is abundant (about 10 mM) in the liver (Chowdhury et al., 2020). When APA overdoses occur, a significant amount of NAPQI is produced, depleting the limited storage of GSH in the cytoplasm and mitochondria. The excessive NAPQI binds to cellular proteins, especially mitochondrial proteins, leading to mitochondrial oxidative stress and dysfunction (Moles et al., 2018; Qiu et al., 1998). In our study, APA decreased TAC and increased TOS.

Induction of NF‐κB overexpression by TNF‐α, IL‐1β, and iNOS may play a role in regulating the liver injury process (Ko et al., 2017). The presence of neutrophils and macrophages in the liver vasculature is crucial in APA‐induced liver injury. Studies have shown that increased neutrophil accumulation is correlated with disease progression, as they infiltrate the liver parenchyma and release cytokines and chemokines. Elevated tissue MPO levels indicate the presence of infiltrating neutrophils, while macrophages also contribute by producing pro‐inflammatory cytokines (Bertola et al., 2013; Liu et al., 2006; Mendes‐Braz et al., 2012). In our study, we found that macrophages increased the expression of IL‐6 and TNF‐α in response to liver toxicity induced by APA. It has been suggested that APA‐induced liver injury leads to sterile inflammation. When cells die, they release damage‐associated molecular patterns (DAMP) molecules, which then stimulate the production of cytokines as a response. This inflammatory response leads to the recruitment of neutrophils and monocytes into the liver blood vessels (Williams et al., 2010).

The FDA approved the use of the antioxidant N‐acetylcysteine (NAC) in 2004 to treat intrinsic liver injury caused by excessive APA intake. NAC is currently the only FDA‐approved APA antidote. NAC acts as a precursor to GSH, reducing the covalent binding of NAPQI to cellular proteins and lessening hepatocyte necrosis. It also reduces the inflammatory response in the liver and improves mitochondrial energy metabolism. However, NAC is ineffective if patients overdose on APA and seek medical help too late, potentially requiring liver transplantation instead (Chowdhury et al., 2020; Craig et al., 2010; Lasram et al., 2015; Saito et al., 2010).

There are studies that indicate the positive impact of alpha‐pinene on liver health. Alpha‐pinene was studied for its effects on cell cycle regulation in liver cancer cells. The treatment led to growth inhibition due to G2/M phase cell cycle arrest. Alpha‐pinene down‐regulated CDK1 and miR‐221 levels, and up‐regulated CDKN1B/p27, γ‐H2AX, phosphorylated ATM, phosphorylated Chk2, and phosphorylated p53 levels. It inhibits miR221 expression, leading to G2/M phase arrest and activation of CDKN1B/p27‐CDK1 and ATM‐p53‐Chk2 pathways, which suppress liver tumor progression (Xu et al., 2018).

Our results show that alpha‐pinene increases TAC levels while decreasing TOS, AST, ALT, ALP, and total bilirubin. Additionally, our findings indicate that alpha‐pinene down‐regulates NF‐kB expression, resulting in reduced IL‐6 and TNF‐a levels.

Drugs and treatments that are effective in humans may never be developed because they fail in animal studies. It's difficult to know how often this occurs because drugs that fail in animals are rarely tested in humans. There have been notable cases where results from animal studies did not hold for humans. One limitation of animal studies is that their findings may not generalize to humans. Therefore, we recommend conducting more comprehensive studies on the beneficial effects of alpha‐pinene in protecting the liver against APA.

In our study, alpha‐pinene demonstrated effects similar to those of silymarin and was effective in preventing liver damage caused by APA. Silymarin is a hepatoprotective agent commonly used to treat liver injuries of various origins. In a study aimed at evaluating the potential beneficial effects of Silymarin (SLM), Balb/c mice were pretreated with SLM at a dosage of 100 mg/kg body weight administered orally once daily for 3 days. Two hours after the final dose of SLM, the mice were given acetaminophen (APAP) at a dosage of 300 mg/kg body weight via intraperitoneal injection, and they were sacrificed at three time points: 6 h (T6), 12 h (T12), and 24 h (T24) later. The results showed that SLM‐treated mice experienced a significant reduction in liver injury induced by APAP, as evidenced by lower levels of AST and ALT enzymes released into the blood and through histological examination of liver tissue. The treatment with SLM also significantly decreased superoxide production, indicated by lower levels of GSSG, reduced induction of heme oxygenase‐1 (HO‐1), diminished nitrosative stress, and decreased activation of JNK (p‐JNK). Additionally, direct measurements of mitochondrial superoxide production in vitro supported these findings. Histological analysis revealed that necrosis was the predominant cell death pathway in cases of APAP poisoning and that pretreatment with SLM could partially prevent this necrosis (Papackova et al., 2018).

One limitation of the present study is the lack of measurement of changes in body fat tissue and food intake in rats, which should be addressed in future studies.

## 5 CONCLUSIONS

According to our study results, alpha‐pinene increased TAC and decreased TOS, AST, ALT, ALP, and total bilirubin. Additionally, it reduced NF‐κB expression, leading to decreased levels of IL‐6 and TNF‐a.

## AUTHOR CONTRIBUTIONS

Conceptualization, methodology, formal analysis, investigation, data collection, writing, review and editing: K.R, A.R, P.Sh, Y.A, M.T; Project Administration: K.R.; Funding Acquisition, K.R. All authors have read and agreed to the published version of the manuscript.

## FUNDING INFORMATION

This study was supported by Shahid Chamran University of Ahvaz, Ahvaz, Iran (NO: SCU.VB1402.50857).

## CONFLICT OF INTEREST STATEMENT

None.

## 6 ETHICS STATEMENT

The study protocol was approved by the Ethics Committee of the Faculty of Veterinary Medicine, Shahid Chamran University of Ahvaz, Ahvaz, Iran (IR.SCU.REC.1402.054).

## Data Availability

Data are available upon reasonable request.
